# Comparative Advantages of Lithotomy and Lithotrity

**Published:** 1849-05

**Authors:** 


*Comparative advantages of Lithotomy and Lithotrity.*—M. Guersant, in one of his late clinical lectures on the *surgical diseases of children* gives the following very interesting table of the comparative advantages and disadvantages of the operation of lithotomy and lithotrity.

*Advantages.*

| Lithotomy. | Lithotrity. |
|---|---|
| 1. Promptness of the operation. | 1. No wound. |
| 2. Always accomplished at one sitting. | 2. No hemorrhage. |
| 3. Complete extraction of the calculi. | 3. Not necessary to confine the patient to bed. |
| 4. No fear of allowing any to remain. | 4. No fear of consecutive inflammation. |
| 5. Applicability of the operation to all ages and to all calculi. | |

According to this table we find the advantages about equal; it is not the same however as to the inconveniences.

*Inconveniences.*

| | |
|---|---|
| 1. Pain. | 1. Many sittings, and longer than in cutting. |
| 2. Danger of hemorrhage. | 2. Pain as in cutting. |
| 3. Wounding the rectum. | 3. Fruitless sittings which does not occur in cutting. |
| 4. Also of the ejaculatory canals. | 4. Pinching of the bladder. |
| 5. Fear of consecutive inflammation. | 5. Consecutive inflammation. |
| 6. Difficulty of extraction in certain cases. | 6. Engorging of small calculi in the membraneous portion of the urethra. |
| 7. Visico-perineal fistulæ. | 7. Sometimes a consecutive operation |
| | 8. Fear of leaving fragments in the *lacunal* that may exist in the bladder. |
| | 9. Fistula. |
| | 10. Unfavorable conditions in which patients are left after the operation and that at some future time it becomes necessary to cut. |
| | 11. The employ of lithotrity, impossible when the calculus is of great size or encysted. |
| | 12. Breaking oft he instrument. |

After comparing these circumstances, we deduce the following conclusions which tally in every respect with our experience: 1st. Cutting should be the method generally adopted in children. 2d. Lithotrity should be employed only in cases where the calculus can be crushed in a single sitting.

In support of these two propositions, the following figures are cited:

Of 42 individuals cut, 34 recovered 8 died; 4 from the operation, and 4 from intercurrent diseases. Of 21 individuals in whom lithotrity had been employed, 18 boys and 3 girls, 12 recovered and 6 died; 2 from the operation, 4 from intercurrent diseases;—3 were afterwards cut.

Thus of the 42 subjects cut for stone, 4 died from the operation; and of the 21 operated on by lithotrity, 2 died from the operation.

